# Efficient branch-and-bound techniques for two-locus association mapping

**DOI:** 10.1186/1471-2105-12-S11-A3

**Published:** 2011-11-21

**Authors:** Karin Klotzbücher, Yasushi Kobayashi, Nino Shervashidze, Oliver Stegle, Bertram Müller-Myhsok, Detlef Weigel, Karsten Borgwardt

**Affiliations:** 1 Machine Learning & Computational Biology Research Group, MPIs Tübingen, Tübingen, Germany; 2 Max Planck Institute for Developmental Biology, Tübingen, Germany; 3 Max Planck Institute for Psychiatry, Munich, Germany; 4 Zentrum für Bioinformatik, Universität Tübingen, Tübingen, Germany

## Background

In this project we want to determine pairs of single nucleotide polymorphisms (SNPs) which have a statistically significant effect on the phenotypic variation of the flowering time of *Arabidopsis thaliana*.

## Material and methods

For a large-scale dataset of over 200,000 SNPs from about 200 individuals together with several phenotypes, published by Atwell et al. [1], we develop efficient methods to find pairs of SNPs which are strongly associated with the phenotype. As an exhaustive search of all possible combinations of interacting SNPs is often unfeasible, even when only considering pairs of interacting SNPs, the challenge is to find methods which avoid an exhaustive search but can still guarantee to find the causal pair. We propose two distinct approaches to efficiently determine the t top-scoring pairs of SNPs.

## Results and conclusions

In the first approach we employ a branch-and-bound strategy to reduce the search space by pruning insignificant pairs of SNPs. Based on this branch-and-bound strategy we develop the two methods fastHSIC and COAT, which use as association measures the Hilbert-Schmidt Independence Criterion (HSIC) [2] and Pearson's correlation coefficient, respectively. The key idea is that we are able to bound the association scores of pairs of SNPs for both methods based only on the association score of one of the SNPs of the pair.

In our second approach we use prior biological knowledge to select a much smaller subset of candidate genes which, according to other findings, affect the flowering time of *Arabidopsis thaliana*. These candidate genes and interactions between them make up a network of 1,452 nodes or genes and 938 edges or gene-gene interactions, and allow us to select a subset of SNPs that lie within or in close proximity to the genes of the network.

Empirical evaluation of our own as well as traditional methods on the original and the reduced dataset shows that both our approaches can greatly reduce the runtime.

## References

[B1] Atwell S Huang YS Vilhjalmsson BJ Willems G Horton M Li Y Meng D Platt A Tarone AM Hu TT Genome-wide association study of 107 phenotypes in Arabidopsis thaliana inbred lines Nature 2010 465 7298 627 631 10.1038/nature08800 20336072 PMC3023908

[B2] Gretton A Bousquet O Smola A Schölkopf B Measuring statistical dependence with Hilbert-Schmidt Norms. Proceedings of the International Conference on Algorithmic Learning Theory J Gen Virol 2005 63 78

